# Perspectives of humanitarian actors on interprofessional care for persons living with diabetes: Lessons from Aleppo, Syria

**DOI:** 10.1016/j.jmh.2025.100355

**Published:** 2025-09-05

**Authors:** Sigiriya Aebischer Perone, Kinda Khamasmie, Ranim Doukki, Claudine Dauby, Catherine Savoy, François Chappuis, Nicolas Perone, David Beran

**Affiliations:** a Health Unit, International Committee of the Red Cross (ICRC), 19, avenue de la Paix, Geneva 1202, Switzerland; b Division of Tropical and Humanitarian Medicine, Geneva University Hospitals, Rue Gabrielle-Perret-Gentil 6, Geneva 1205, Switzerland; c International Committee of the Red Cross, Damascus, Syria; d International Committee of the Red Cross, Aleppo, Syria; e International Committee of the Red Cross, Kinshasa, Democratic Republic of the Congo; f Division of Tropical and Humanitarian Medicine, Geneva University Hospitals, and Faculté de Médecine, Université de Genève, Rue Gabrielle-Perret-Gentil 6, Geneva 1205, Switzerland

**Keywords:** Interprofessional, Care continuity, Diabetes, Chronic diseases, Humanitarian, Patient care team

## Abstract

**Background:**

Proper management of persons living with chronic diseases including diabetes requires collaboration between different health care providers. Yet in humanitarian settings, care is essentially provided by health services organised in silos, without coordination and inclusion of people living with various conditions. Interprofessional teamwork could be a means to address this, but this approach is rarely implemented in such contexts.

**Methods:**

Semi-structured interviews were conducted with a purposive sample of 12 health care professionals from the International Committee of the Red Cross in Syria and in headquarters Switzerland, to explore their perceptions and experiences of factors influencing the work across professional teams. Qualitative analysis was conducted using an inductive and deductive thematic approach.

**Main findings:**

The perspectives of humanitarian actors on interprofessional care for persons living with diabetes in a humanitarian setting identified four key factors that influence interprofessional collaboration: i) person focused inclusive leadership to overcome competing demands and competition between health teams, ii) reorganisation of health services with a clear structure, appropriate resources and processes for interprofessional collaboration, iii) commitment and openness of the individual health professional to work collaboratively and iv) the key role and capacities of people with diabetes, who need to be included as team members. These four elements interact and need to come together to ensure successful interprofessional care.

**Implications:**

The findings advocate for an inclusive leadership of the interprofessional team, reorganisation of work, empowerment of all team members, and inclusion of people with a chronic condition in the team. Health care providers are thus encouraged to support interprofessional care to address the needs of people living with a chronic condition in a humanitarian context.

## Highlights

• Interprofessional care for PLWNCDs in humanitarian crises is feasible and requires strong commitment and support from all involved.
• Institution's high-level managers must be the leaders of the change processes to enable building an interprofessional team.
• Care must be re-organised with allocation of appropriate resources, definition of roles and responsibilities.
• Individual team member contribution and their commitment is key.
• Patients need to be involved the interprofessional team for informed shared decision making and appropriate management of their condition.
• The four dimensions of interprofessional care can be integrated in the WHO’s health system building blocks for scale up.

## 1 Introduction

Current estimates from the World Health Organisation (WHO) are that 74 % of deaths worldwide are attributable to noncommunicable diseases (NCD), mainly in the form of cardiovascular diseases, chronic respiratory diseases, diabetes (DM) and cancer (World Health Organisation, 2023). NCDs are chronic in nature, interact with mental health (Willis et al., 2023) and share common risk factors (Stein et al., 2019).

In humanitarian settings, NCD prevention, treatment and management is challenging due to many factors such as: historical health care focus on trauma, acute care and single interventions (Boulle et al., 2019); shortage or loss of infrastructure, challenges in accessing healthy food, medicines and supplies, as well as psychological distress (Charlson et al., 2019; International Diabetes Federation 2023); recent shifts in the humanitarian environment with protracted crises (International Committee of the Red Cross 2021); decreased funding of health care systems (Witter et al., 2020) and shortage of trained health workforce (World Health Organisation 2021) exacerbated due to attacks, workload, economic crises and poor working conditions (Elnakib et al., 2021). All these factors restrict patients’ ability to adequately manage their condition, and health professionals’ ability to care and monitor people living with chronic conditions. Interruption in access to medicines and care puts people living with NCDs (PLWNCD) at risk of complications or flare ups (Hayman, 2015; Slama et al., 2017; Ngaruiya et al., 2022), as seen in diabetic foot complications requiring lower limb amputation (Barth et al., 2021).

In stable settings standard practice is to deliver NCD prevention, treatment and management using an interprofessional approach (Antoni, 2010), addressing physical, mental health care and rehabilitation needs. Interprofessional care of persons living with chronic conditions may be demanding, as it requires professionals of different health programs, services or teams and hierarchies to collaborate, reorganise themselves and possibly work across competing priorities (Boulle et al., 2019; World Health Organisation, 2018)**.** Interprofessional care reduces the burden on health care providers (World Health Organisation, 2018), improves accountability to affected persons (International Committee of the Red Cross 2019) and quality of care with a positive impact on diabetic foot outcomes (Buggy and Moore, 2017). Yet, evidence on best models of care in humanitarian contexts remains scarce (Jaung et al., 2021) and are currently explored in the Middle East by different humanitarian actors (Ansbro et al., 2021; Willis et al., 2023; Truppa et al., 2023).

According to the United Nation’s report, as of 2023, the conflict in Syria displaced internally 7.2 million people (UN Office for the Coordination of Humanitarian Affairs 2024). It resulted also in an estimated 50 % of health staff having left the country, a financial crisis (UN Office for the Coordination of Humanitarian Affairs 2024) with the minimum expenditure basket (measuring cost of living) rising by 21 % in a year (Humanitarian programme cycle 2025), an increase of 400 % in medicine prices since 2019, and shortages of oral medicines, insulin and consumables (World Health Organisation, 2016). DM prevalence in Syria was estimated to be 12.6 to 14.8 % in 2011 (Alali 2022) (Alali and Afandi, 2022). According to the International Diabetes Federation (IDF) atlas, there were about 1′500′000 adults reported living with diabetes in 2020 (International Diabetes Federation, 2021), potentially facing, due to the armed conflict, psychosocial trauma, food insecurity, limited access to health services, medicines and testing supplies, with an increased need to self-manage their condition (Khan et al., 2019). In Aleppo, the reported prevalence of diabetes in 2010 was 15.6 %, higher than the country prevalence (Albache et al., 2010). Besides health needs, people living with diabetes (PLD) in Aleppo may face other challenges such as being several times displaced, have missing family members, and other competing priorities (Alali and Afandi, 2022; Khan et al., 2019; Humanitarian programme cycle 2025).

To address the needs of PLD, the International Committee of the Red Cross (ICRC) (International Committee of the Red Cross 2017) supports since 2017 selected Ministry of Health’s (MoH) Primary Health Care (PHC) clinics, DM specialised centres serving both adults and about 800 children with type 1 DM, hospitals and Syrian Arab Red Crescent (SARC) clinics. In addition, the organisation set up an ICRC Mental Health and Psychosocial Support (MHPSS) program and a Physical Rehabilitation Centre (PRC) in Aleppo. This centre provides services to 1′000 persons per year, with 65 % of service users living with an amputation, caused in 20 % by DM (Khamasmie et al., 2021). Aleppo was chosen as the case study location, as a representative site of a humanitarian setting with, in 2021, 70′000 internally displaced people in the Governorate (REACH 2021), a high number of PLD living with an amputation attending the PRC (Khamasmie et al., 2021) and the presence of ICRC PHC, MHPSS and PRP teams working at the same location. Yet, while ICRC’s MHPSS and Physical Rehabilitation Program (PRP) teams were co-located, no formalised system existed for comprehensive management of PLD and their inclusion as team members.

## 2 Material and methods

The objective of this qualitative study, was to investigate factors influencing interprofessional collaboration. This paper presents the results of interviews with ICRC’s health staff as a first step of an ongoing program to improve care and support offered to PLWNCD and diabetes within ICRC’s supported health facilities in Syria and elsewhere.

### 2.1 Study design

Semi-structured interviews with a purposive sample of 12 health professionals were conducted to explore their perceptions and experiences of factors influencing the work across health programs and professional teams within the different dimensions of interprofessional care.

### 2.2 Setting and participants

ICRC’s health set-up at headquarters and in the field (delegation and sub-delegation, e.g. in Syria Damascus and Aleppo) consists of six programs (first aid and prehospital care; PHC; hospital; MHPSS, physical rehabilitation; health in detention) (International Committee of the Red Cross 2015), with program specific priorities and reporting lines to each program’s hierarchy. Staff at headquarters provide strategic guidance and support to those based in the field where programs are implemented. Direct supervision and support of field teams at sub-delegation level is then given by the field staff based in the delegation. In Aleppo, while the MHPSS and PRP teams were directly involved in patient care, the PHC team supported MoH and SARC PHCs, as well as a MoH specialised diabetes centre. Although the setting was appropriate for standard care of PLD, interprofessional care provision, would require medical teams with different hierarchies to closely collaborate, reorganise themselves, work across professions and possibly competing priorities.

Study participants were selected by applying a purposive sampling strategy (Nyimbili and Nyimbili, 2024), to reflect the geographic, program and professional characteristics of ICRC health staff involved in DM care, working in the PHC, mental health or physical rehabilitation programs in Syria and in headquarters. Participants were equally distributed across the three programs, with one national and one international staff member from Aleppo (six participants) and their hierarchical program counterparts in Damascus and in headquarters (one national or international staff member per program). The purpose of the study was presented during a health meeting in Geneva and Syria and participants were invited via e-mail. Inclusion criteria were ICRC international and national staff members; English speaking; working in the field of DM care in Aleppo, Damascus and Geneva; and belonging to the professions and programs of physical rehabilitation, mental health, and primary care. Exclusion criteria were persons not belonging to health staff or not involved in DM care.

### 2.3 Data management and analysis

Semi-structured interviews were conducted between December 2021 and January 2022 by the same interviewer with participants located in Syria (Aleppo and Damascus), and headquarters (Geneva Switzerland) by using teleconferencing tools (Skype or Teams). All interviews lasted between 45 and 75 min. Written, informed consent was received from all participants. Interviews were audio-recorded, anonymised and transcribed verbatim. De-identified transcripts were imported into MAXQDA® for coding and extracted on an Excel file for analysis. Data was analysed using an iterative approach of inductive and deductive analysis. i) The initial deductive analysis coding was guided by the conceptual frameworks of interprofessional collaboration, job demands and resources, team and professional identity and role of the patient. ii) Unexpected findings and new themes were elicited by inductive analysis. Their robustness was then explored in existing literature and with regards to the research question and included in the coding if robust. iii) The coding template was iteratively adapted to emerging themes.

### 2.4 Ethics approval

After completion and submission of ICRC’s ethics review form, the study received ethical clearance from ICRC’s ethics review board “Centre for Operational Research and Experience” (CORE) (International Committee of the Red Cross 2024) under the title “Factors that enable individuals to think beyond the silos in medical settings and live the continuum of care. Interprofessional collaboration of ICRC health teams for diabetes care in Aleppo, Syria”. The study was also approved by ICRC’s management in Syria and health unit in Geneva. We required and obtained consent from all participants prior to the interviews and got the approval of the participants to cite their quotes.

### 2.5 Theory

Based on preliminary experience and a literature review, an interview topic guide was developed bringing together the following different frameworks (See Table 1):

i) the framework on interprofessional collaboration proposed by Mickan and Rodger (Mickan and Rodger, 2000) summarizing effective team work in three categories of organisational structure, individual contribution and team process;
ii) a model on moderating roles of team and professional identity in interprofessional effectiveness and protective routines where Liff (Liff and Wikström, 2015) highlights the fact that units composing teams use “protective routines” to reduce the threat to their professional identity and that this leads to “compartmentalisation in multi-professional teams in healthcare” that ”inhibits productive interaction”, and “also influences potential affording situations negatively”. The task of the team leader is to reduce the threat to their professional identity and creating affording situations. Competition between teams and challenges linked to professional identity are also presented by Mitchell (Mitchell et al., 2011);
iii) the job demands and resources model (JD-R Model) (Bakker and Demerouti, 2007) assuming that “job strain develops – irrespective of the type of job occupation – when (certain) job demands are high and when (certain) job resources are limited”. In contrast, work engagement is most likely when job resources are high, including if facing high demands. When demands are high, people then seek to retain and protect what they value (eg. the “protective routines” as described by Liff (Liff and Wikström, 2015), and experience stress in relation to loss of potential or actual resources). The reorganisation of health services was evaluated by using the JD-R Model (Martinussen et al., 2017), where job demands were associated with burnout and job resources with engagement and job satisfaction. The interaction between workload and collaboration showed that good collaboration moderates or buffers the effect of job demand on quality. The reorganisation increased collaboration, leadership and quality with higher engagement than observed in the general population. It had no effect on workload, autonomy, engagement, social support and job satisfaction and decreased work conflict and cynicism compared to the general population.

**Table 1:** Topics explored in the interview guide.

Key area	Dimensions
Organisational structure	Purpose
	Goals and responsibility
	Culture
	Professional identity threat, team identity and protective routines
	Tasks
	Roles
	Leadership
	Members
	Resources
	Demands
Individual contribution	Self-knowledge
	Trust
	Commitment
	Flexibility
Team process	Coordination
	Communication
	Cohesion
	Decision taking
	Conflict management
	Social relations
	Performance feed-back
Role of person with condition	

The study also explored the role of the person living with the condition.

## 3 Results

### 3.1 Demographic data

All twelve contacted ICRC health professionals in headquarters and in Syria accepted to be interviewed and recorded. In all, ten individuals were interviewed alone, while two from the same program in Aleppo (a national and international staff member) wished to be interviewed together for convenience reasons linked to access to stable internet and timing. Table 2 summarises participants’ characteristics showing an equal distribution of participants across the programs, and a predominance of international versus national staff members, corresponding to the distribution in the locations, with more international staff based at headquarters and in central field sites (delegation level).

**Table 2:** Characteristics of participants interviewed (n=12).

**Characteristic**	**Number**
**Gender**	
Male	2
Female	10
**Contractual status**	
International staff	8
National staff	4
**Profession**	
Nurse	3
Doctor	2
Physiotherapist	4
Psychologist	3
**Programs / Teams**	
Primary health care	4
Physical rehabilitation	4
Mental health	4
**Location**	
Headquarter	3 – 3 international staff members
Field	
Damascus	3 – 1 national and 2 international staff members
Aleppo	6 – 3 national and 3 international staff members

The emerging themes were put forward to provide further ground for interprofessional care and (future) interventions. They are related to the health system with leadership skills and behaviours, organisation of the interprofessional team, characteristics of individual team members and to persons living with a chronic condition as shown in Table 3.

**Table 3:** Emerging themes.

**Person focused inclusive leadership skills and behaviours**
Create a common understanding and a shared vision of interprofessional collaboration overcoming competing demands and competition between teams and challenges linked to professional identity
Build trust: be listening, self-aware, create an appropriate culture, be consistent and accountable
Show consideration of all involved: acknowledge the importance of the role of every team member and include them
Empower team members: allow for mistakes and learn from them; give space and decision latitude
Show organisational commitment
**Organisational factors**
Set an organisational structure for the team, which has to be co-located, managed by a focal point with established procedures for interprofessional collaboration including documentation, provision of adequate resources, specified roles and tasks
Manage team process: coordinate, communicate, share information, take decisions, hold team meetings, manage conflict, provide performance feedback
Enhance collaborative skills and mutual learning through exchange of knowledge and capacity building activities
**Characteristics related to the team members**
Have a common understanding and a shared vision
Trust colleagues and favour social relationships
Be motivated to work collaboratively
Demonstrate the following individual characteristics of self-awareness, flexibility, listening skills, openness to learn
Apply the previous experience of interprofessional collaboration
Be committed to achieving quality outcome
**Patient related factors – elements to include in patient-centred interprofessional care**
Provide a tailored response to patients such as information, medicines, devices, and financial support based on their needs and capacities.
Raise awareness for patient’s understanding of their health conditions; share responsibility with the patient to take his/her own decisions (shared decision making)
Build trust and facilitate commitment

### 3.2 Theme 1 – Leadership factors

We need to distinguish between two different levels of leadership. The first institutional level has a supporting role in building and organising interprofessional team dynamics. The institution's high-level managers must also be the leaders of the change processes that enable team building. This must be reflected not only by advocacy on interprofessional teamwork, but also by the introduction of mechanisms and tools to enable the health professionals to implement it. While ICRC’s traditional health focus was on care for weapon wounded people, care for PLWNCDs was formalized in the 2014–2018 health strategy (International Committee of the Red Cross 2014). Thereafter, NCD care started to be integrated into the different health programs, requiring hierarchical support in implementing a continuum of care and interprofessional approach (International Committee of the Red Cross 2020). This was highlighted by all participants, who mentioned the need of organisational commitment and support by managers, be it from the different hierarchy levels of health or ICRC management, in addition to the importance of leaders setting an example.

“And the management is willing, is considering…. It’s very hard if you don’t have the support to actually implement it (the interprofessional care)… (PHC)

“…leaders and leadership, it means somebody who sets the example, practice what they preach…. If people are not consistent with actions, they lose their credibility. ….…organizational culture, that really…encourages this working in teams, not in silos.” (MHPSS)

This institutional leadership should be given concrete expression by formalizing interprofessional collaboration in the job description and evaluation of staff, as stated by one participant in a supervising position,

"…Should be added for example, in the job description or an evaluation of everybody… how well are you promoting interdisciplinary or interprofessional work…." (PRP)

The second level of leadership takes place within the team. By definition (Mickan and Rodger, 2000), a team exercises shared leadership, expressed by an "equity" of representation and decision-making, whatever the individual's function or role. To be put into practice, team leadership requires a good understanding of each person's skills, and mutual trust, enabling difficult decisions to be taken, implemented and assumed as a team.

Interprofessional team leaders should empower team members and support them when carrying out their tasks, facilitate learning from experience, share successes, give space and decision latitude, jointly bear mistakes, and involve all in the provision of interprofessional care. The central theme of trust building between the different programs’ professionals was underlined by field and headquarter participants. While they are aware of their own program objectives and capacities in each site, they may not be aware of the ones of other programs and sites. Trust can be built by knowing each other personally and their professional capacities and program orientations. Mutual knowledge creates a safe and respectful environment where people can expose their views, facilitating shared decision making.

"You're not well aware of what others are doing, and what are their competencies, then you will not reach out to the others…I think this is key to know well what others are doing …, to learn more about their own profession…People need in order to work well together, to trust each other. So, if you don't trust the other program or the other person, then it will be difficult to work together." (PRP)

The task of the leader is to prioritise the integration of the different professionals in the interprofessional team giving the same importance to all. Indeed, different health program’s strong identities, may favour work in silos, if they feel threatened in their professional identity when working in an interprofessional team. Each program reports to different hierarchies, with dual accountabilities and may have competing demands and priorities, that could inhibit constructive interactions. Participants from the PRP and MHPSS professions at headquarters and in the field reported competition between the different teams for resources leading to work in silos.

“Because when different teams (ICRC health programs) with different skills, capacity are working together, there is a tendency to do like a competition. I want to do more, to be the best, to provide the best services. I want to have more results. Then they may create the tendency to work in silos, because people will focus only on their result, on their objective.“ (MHPSS)

In addition, some participants reported a feeling of not being considered as an “equal team member” and asking for acknowledgement and valuing of their profession and work. Therefore, leaders should support employees involved in interprofessional dynamics by highlighting and recognizing their efforts and the importance of their program.

*“*…the importance of rehabilitation within health, then we would feel more included…. Everybody needs to feel valued, that his contribution is valued within the team and not left aside like this*…*." (PRP)

### 3.3 Theme 2 – Organisational factors

The implementation of interprofessional team dynamics requires the setup of clear team processes and an organisational structure, as mentioned by the participants. This encompasses co-locating the different professionals, setting up coordination and communication mechanisms, such as “physical” and distance meetings managed by a focal point, definition of clear roles and responsibilities, creating shared tools and defining processes for conflict management.

Regarding coordination, communication, information and experience sharing, participants highlighted the importance of joint meetings (general on organisation of care and clinical), field trips and assessments. Participants suggested that during meetings representatives of the different professions would take turns in speaking and taking notes, allowing for everyone to be actively involved and participating. During clinical meetings, situations related to their work were presented in a structured way including at the end a question-and-answer section. These meetings facilitated knowledge exchange, capacity building and mutual learning, where for example MHPSS professionals, covered topics of communication, empowerment of patients and mental health. Moreover, if PLD would entrust them with relevant clinical information involving other professionals, they transmitted it directly to the concerned professional. In case of general information relevant to other professionals, they reported it during the general meetings, held to coordinate and organise interprofessional care, enhancing collaborative skills. In absence of having the possibility of holding face to face meetings due to COVID-19 restrictions, participants proposed alternative communication channels such as e-mails and boards with essential information.

“… comprising representatives from all the three (professional teams), MHPSS, us health and PRP. So, we meet every week, as response to what is pending, what needs to be done, updating files, and setting guidelines…. We are working together within a team…. and there is one who is responsible." (PHC)

All interviewees mentioned that members of an interprofessional team must recognize their specific roles, responsibilities, and accountability within the team, allowing their effective contribution and avoiding conflict.

"Each team member must have clear roles and how that role is placed within the team as well. Everybody must understand what they're supposed to do and how it relates to the next person, to the next team member or to their managers." (PHC)

The interoperability of information systems is crucial, given that each professional tends to favor the use of a specific “patient” tool. This diversity of tools makes the sharing of information between these platforms particularly challenging. Therefore, participants working in the field mentioned the need to establish a common tool to facilitate exchange of information.

"We have many tools to assess, to evaluate, monitor, let's say data collection and monitoring tools. But these tools are developed at each department… We should have one tool, that includes all those aspects to keep in loop of all these aspects (of PHC, MHPSS and rehabilitation) ." (MHPSS)

Conflict resolution between the different programs or teams was seen by field and headquarter participants as needing to be managed by collecting the point of view of all concerned persons and discussing the different opinions openly within a solution-oriented and patient/person centred approach.

"Sometimes you can also have the solution coming up by team members and this could help to serve the issue…. just to have the point of view of everybody and what's the problem behind, what is the feeling of the team members." (PRP)

### 3.4 Theme 3 – Factors related to the team member

Every team member is individually contributing to the interprofessional team. Individuals must adhere to the “new way” of working together and be committed to work beyond their own program to address the needs of PLWNCD in a comprehensive way. This may be seen as additional workload and diverting resources from program specific objectives. To align efforts, interviewees acknowledged the importance of having a common understanding and a shared vision of the interprofessional collaboration’s goals, and on how contributions from the team members allows the person with the chronic disease to be in the centre.

"It’s a priority…We have to understand that beneficiaries (are) in the centre and all of us we are a team…I think this is the most important thing…that when we do something, we cannot work in silos… we have to change the mindset." (MHPSS)

To be able to work efficiently with other individuals and professionals within an interprofessional team, many participants featured trust, good social interaction, respecting the different values and contributions of all. Working as part of a team requires to question one’s autonomy and lose a certain amount of control, by letting other members contribute to the decision-making process. Creating and maintaining trust and good interpersonal relations, allows to reach out to the different team members, sharing information and addressing together challenges. It’s by working as a team with the different competencies put together that we can improve our skills and the quality of our care.

"Establish a good relationship…to be seen truly that you are not God, you don’t know everything, that you have limitations, and you are open to learn… sit down, exchange knowledge and discuss together…are necessary to set the ground for a good teamwork." (MHPSS)

"…and then to feel confident enough to also raise up maybe some concerns or something that is not working well or that the person is not confident with, you know, and ask also others, maybe you can help me on that." (PRP)

### 3.5 Theme 4 – Person living with the condition related factors

Involving PLWNCD in the team dynamics was recognised as essential and mentioned by the field teams. This was not yet consistently applied in the different health programs in Syria and elsewhere. Patient involvement in their care plan would enable consolidating the findings of the different assessments, and defining care objectives that incorporate their preferences, capacities and priorities. Patients’ perspectives, including information on access to basic needs such as food and shelter, faced challenges of being displaced and having lost loved ones impacting possibly their mental health, and feedback on proposed treatment, are key to successful management of their condition.

"We put the focus with the patient, get their feedback deliberately to know what we could do better, to help us improve the quality of services that we are providing." (PHC)

"The role of the patient is the factor what will influence our success…Because we can do what we can, we can provide quality of services. We can work together as a team, but if the patient is not involved and understands the process…, it will be very difficult for us to reach the expected objective result." (MHPSS)

Persons living with chronic conditions need to trust the health professional to provide all the necessary information. They need to be empowered to understand and adhere to the treatment. When the PLD is part of the team it's easier to recognize his or her strengths and limitations. In case of financial or social problem impacting on care, interprofessional collaboration allows to adapt strategies and tailor them to the identified needs and capacities and co-develop a feasible and acceptable care plan.

"Free of charge treatment would be easier for them, than changing their lifestyle. For example, you will advise any diabetic patient not to eat bread, and this would be not acceptable for them at all, because people are not getting enough food. They are depending on bread sometimes, so this is an example and advice that they cannot follow." (PHC)

Many participants acknowledged that in Aleppo, PLD were frequently not aware of their condition. Therefore, they emphasised awareness raising as an initial essential step, leading then to empowerment of PLD and shared responsibility and decision making.

"I would not be surprised that they are not even aware about they have diabetes….then they don't know, so they don't feel responsible. I believe that when we do any kind of awareness (rising), we have to make it so clear that’s a co-responsibility. Unless the patient is equally responsible, unless a patient is equally into it, it will never work…. And we have not to forget that 10 years of conflict has really pushed back their mentality towards taking care of themselves." (MHPSS)

**Fig. 1:**
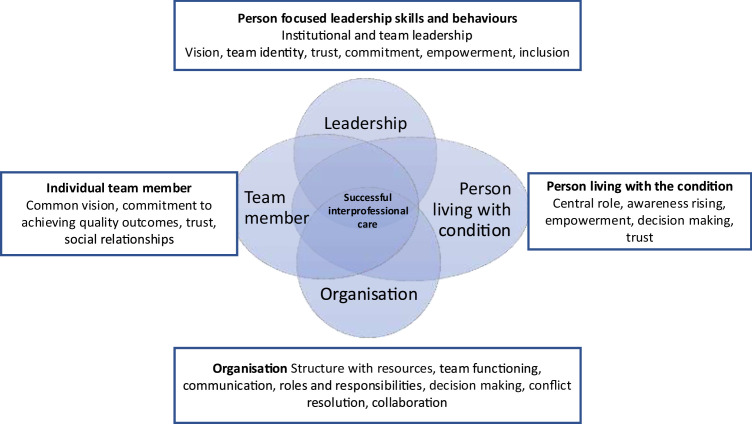
Factors favouring interprofessional care and their interaction.

The four themes of person focused leadership skills and behaviours, organisation of care, characteristics of the individual professionals and persons living with chronic conditions are overlapping and need to come together in to ensure optimal interprofessional care as shown in Fig. 1.

## 4 Discussion

The exploration of the participants’ perceptions of factors influencing interprofessional collaboration for PLD in a humanitarian setting, was based on the framework of effective interprofessional collaboration (Mickan and Rodger, 2000), the job demands and resources model (Bakker and Demerouti, 2007; Martinussen et al., 2017; Lequerre, 2013), completed by professional team identity (Liff and Wikström, 2015; Mitchell et al., 2011). Findings from the study highlight the interaction of four dimensions: inclusive leadership skills and behaviours; factors related to care organisation; commitment and characteristics of individual team members; and central role of PLD (including capacities and needs of individuals living in a humanitarian context).

Firstly, leaders of interprofessional teams should be consistent, coherent in words and action, create a common understanding with a shared vision of interprofessional collaboration and share the knowledge of the goals and strategies of the institution (Lequerre, 2013; Leggat, 2007). Competing demands (Bakker and Demerouti, 2007) and priorities (Boulle et al., 2019), competition between health programs and challenges linked to professional identity (Mitchell et al., 2011) are acknowledged as barriers to interprofessional collaboration. In a humanitarian context, competing demands and priorities may be exacerbated due the challenges in provision of essential goods such as food, shelter and continuous supplies in medicines and diagnostic tools, in addition to needing to modify the treatment as reported for PLD in Syria (Alali and Afandi, 2022; Khan et al., 2019) and providing care to displaced persons (Willis et al., 2023). Competition between health programs and strong professional identities were observed as some professionals used mitigation measures of “protective routines” to reduce the “threat to their professional identity” and “loss of professional values and status” (Liff and Wikström, 2015). In addition, several individuals mentioned their feeling of lack of consideration of their role and not being valued as “full” team members. Therefore, as described by Smith (Smith et al., 2020), leaders should be “inspiring and motivating”, empower individuals and show consideration of all involved by acknowledging the importance of the role of every individual and by raising awareness on the specificities and complementarity of the different professionals. This leadership behaviour favours an inclusive culture, where equal importance is given to all team members, power is shared and individuals are respected, empowered and treated with fairness (Diamond, 2019). As mentioned by Lawton (Lawton and Páez, 2015), ethical leadership dimensions of acting fairly and with consideration of others, influence positively both individual’s and group behaviour, satisfaction and performance. In addition, favouring decision latitude, autonomy and good social relations (Bakker and Demerouti, 2007; Lequerre, 2013) mitigate the demands created by interprofessional collaboration. These person-focused leadership skills and behaviours (Smith et al., 2020) allow leaders to build an overarching “new interprofessional team”, encompassing all professionals, where all professions are acknowledged as essential and equal to improve the care of PLD. In addition, regular feed-back on interprofessional collaboration in the performance review were suggested as facilitators, as described by Smith (Smith et al., 2020). Mickan (Mickan and Rodger, 2000) proposes also “team-based incentives that are contingent upon the whole team’s performance”.

Secondly, to reach effective interprofessional collaboration, an interprofessional team must be built, with a well-defined organisational structure and clear team processes. In Syria, an interprofessional team composed by professionals of PHC, MHPSS and PRP was established in Aleppo. Working in silos was mitigated by nominating a team leader or focal point, coordinating the different professionals involved in diabetes care both in Aleppo and Damascus. In a similar health set-up providing care to Syrian refugees and Lebanese in northern Lebanon, the need of defining a focal point was also identified as a key element of provision of interprofessional care (Truppa et al., 2023). Yet, the role of this focal point was to support patients and families navigating the health system, whereas in the current study, the role of the focal point is to coordinate the different teams and act as a team leader. Other organisational factors of tasks, roles and responsibilities were defined, minimising ambiguities about their work (Lequerre, 2013) and “inconsistency between professional's role and how others perceive it” (Mickan and Rodger, 2000). Time, space (colocation of MHPSS, PRP and PHC programs and professionals) and additional material resources were allocated for interprofessional care of PLD. Regular interprofessional team meetings were introduced. These meetings can have the different goals of i) organisation of care (Makic and Wald, 2017), ii) information sharing, iii) integrated patient management, iv) shared decision making and v) capacity building (Sørensen et al., 2018). Up-skilling and training of care providers in diabetes care is key in humanitarian settings where diabetes care may be provided by health professionals with little experience in DM management (Khan et al., 2019; Alali and Afandi, 2022). Interprofessional trainings and team meetings favour peer exchange, strengthen collaboration, trust, mutual knowledge, and respect of all team members (Sørensen et al., 2018; Szafran et al., 2019), who recognise and value their contribution to the team (Mickan and Rodger, 2000). Likewise, experience of success can be shared. Team meetings strengthen team accountability (Mickan and Rodger, 2000) and build up the identity as an interprofessional team providing care for PLD (Liff and Wikström, 2015). In addition, shared patient management tools (Makic and Wald, 2017; Sørensen et al., 2018) started to be developed, facilitating exchange of information and continuity of care. A robust information system was also found as an essential element of interprofessional care provision in northern Lebanon (Truppa et al., 2023).

Thirdly, the role and contribution of individual team members emerged as significant. Team members have to adhere to the vision and goal of interprofessional patient centred care and believe “that the team is the best way to deliver this coordinated care” and cooperate around the PLD in line with their professional values and standards (Mickan and Rodger, 2000). Trust and respect, interpersonal and social relations between the team members are resources and facilitate working collaboratively (Bakker and Demerouti, 2007) and buffer the effect of increased job demand due to reorganisation of care (Martinussen et al., 2017). The different team members have to “develop confidence in each other’s competence and reliability”, acknowledge and “respect another’s skills and expertise, recognise and appreciate the unique skills and contributions of each other to coordinated patient care” (Mickan and Rodger, 2000). Factors or characteristics related to the individual such as self-awareness, being flexible, listening to others with openness to learn and having a previous experience of interprofessional collaboration were frequently mentioned and can be found elsewhere (Mickan and Rodger, 2000). The latter appears to be a strong facilitator of interprofessional collaboration when this experience can be shared and applied (Makic and Wald, 2017). Commitment of team members to work collaboratively (Makic and Wald, 2017) beyond their professional roles and their engagement supported by their managers promotes interprofessional work and quality outcomes (Leggat, 2007) .

Finally, effective interprofessional collaboration such as team based care advocated for by the World Health Organisation (World Health Organisation, 2018) should include the patient, here the PLD, as partner and “full” team member with an active role (Devi et al., 2020), who is able to self-manage his condition (Ansbro et al., 2021; Khan et al., 2019; Truppa et al., 2023). While all participants acknowledged the central role of PLD and importance of their involvement in care, this was not yet done in a systematic way. Many PLD were not even aware about their physical medical condition or mental health issues before consulting. They considered them as “normal”, while a recent cross-sectional study in Syria showed a good to moderate understanding of diabetes and hypertension by the Syrian population (S Swed et al., 2023). The difference observed in our study could be explained due to low socio-economic status of the persons attending ICRC supported services. Raising awareness and patient therapeutic education (Correia et al., 2023) would allow PLD understanding their health conditions, being empowered, taking responsibility, making their own informed choices (Devi et al., 2020) and prioritise their health along with other competing priorities related to their life in a humanitarian crisis setting. In addition, the use of brochures, Short Message System (SMS) or the media have been proposed by the participants. Health promotion must be adapted to the challenges individuals are facing in a humanitarian context with an increase of mental health distress (S Swed et al., 2023), requiring a tailored individualised response to be pertinent. In a humanitarian context, in addition to providing subsidised medical care, consultations and follow-up, access to health facilities, medicines and medical devices needs to be facilitated and financially supported (Willis et al., 2023). Good communication skills of health care workers (Van Dongen et al., 2016) build trust and encourages patients disclosing information pertinent to their health and living conditions, which in turn contributes to the discussion and proposal of adequate care and finally shared decision making between health professionals and patients. This is facilitated and influenced by the interprofessional team, where decisions are also taken jointly (Légaré et al., 2011). Yet, decisions may still be taken centrally in challenging social and humanitarian contexts at the level of the health system.

### 4.1 Strengths and limitations

Although the importance of interprofessional care is well established in many countries for the management of PLD or PLWNCD, interprofessional collaboration remains scarce in humanitarian settings, where care is frequently provided by different health services in silos*.* Here we have focused on the different ICRC health professionals’ perceptions. The results of this study, in addition to further investigations about perceptions of care providers and PLWNCD could serve as basis for the joint development of acceptable, feasible and sustainable interprofessional care, building a shared understanding and intervention strategy for quality patient centred care and services in humanitarian settings.

We acknowledge as limitation the possibility of a selection bias of participants, selected based on their involvement in projects providing care for PLWNCD, and diabetes in particular. The relatively small sample size of the study may limit generalisation and external validity of the findings. Yet, Hennink demonstrated that “saturation can be achieved in a narrow range of interviews (between 9 and 17)...; ; , particularly in studies with relatively homogenous study populations and narrowly defined objectives” (Hennink and Kaiser, 2022), which was the case of our study. We chose individuals to reflect ICRC headquarter and field characteristics of the different health professionals, which was confirmed during field visits in the Middle East. Another limitation might be that a non-Arabic speaker and headquarter-based health professional interviewed the participants. This may impede participants giving negative statements about headquarters or the management and limit interviews to English speakers. Yet, all ICRC headquarter and Syria staff members of the PHC, MHPSS and PRP professions were fluent in English. They held different positions and were of diverse backgrounds (leader or PHC, MHPSS, PRP international or national health professionals). The interviews were conducted with health care workers, providing information about PLDs role and needs as best proxy, as PLD or PLWNCD interviews were not feasible within the scope of this research. Previous research in Lebanon on Syrian refugees and Lebanese living with a NCD and caregivers, reported that they experienced challenges in accessing affordable medicines, lab testing, routine care and in navigating the health system. They also mentioned the importance of family support and stress related to conflict and displacement (Truppa et al., 2023). Similarly in Iraq PLWNCDs faced difficulties of living their condition with emotional and practical impacts including challenges in continued access to medicines and of following healthy diet advice (Ansbro and Schmid, 2025). PLWNCD or PLD interviews in Syria would improve the identification of patients’ challenges of living with a chronic condition in country facing ongoing hostilities, restraint resources, economic decline (Humanitarian programme cycle 2025), beliefs in disease causation and treatment, along with competing priorities and their strengths (Willis et al., 2023). Putting the patient in the centre as partner would also facilitate tailoring better the health services and response to their perceptions, needs, capacities and their involvement in decision making and care.

The situation in Syria continues to evolve with persistent humanitarian response and health needs. According to the humanitarian response priorities for January to March 2025 “NCDs contribute to 50–70 % of all mortality, exacerbated by limited access to services and treatments for these conditions” (Humanitarian programme cycle 2025). Thus expansion of access to interprofessional health services for PLWNCDs along the continuum of care as done in Aleppo could prevent avoidable complications and deaths. This paper’s four dimensions for effective interprofessional care in a humanitarian context of leadership, organizational dynamics, team member contributions, and patient involvement, can be applied to the WHO’s health system building blocks (World Health Organization 2007) with an addition of patients and the community, as shown in Table 4. For scale up, health care managers in other humanitarian contexts, organisations or in LMIC are encouraged to integrate and adapt these interprofessional care dimensions to their setting to facilitate interprofessional care for PLWNCDs.

**Table 4:** Health system inputs.

**Leadership and governance of the health structure**	• Create a shared vision of interprofessional collaboration • Allocate appropriate resources (financial, human, technical, time) • Set an example by working collaboratively across teams at all levels • Establish an appropriate management structure (under the leadership of a focal point or team leader) • Give autonomy to the teams and empower all team members (including patients)
**Financing**	• Facilitate access to care by defining vulnerability criteria for subsidisation of care • Subsidise care (consultations, medicines and consumables, laboratory and other exams, medical devices, transport) for PLWNCDs • Coordinate with other actors and stakeholders (eg for food assistance) for patients with chronic conditions (integration and continuum of care beyond health)
**Service delivery**	• Co-locate medical, rehabilitation and mental health and psycho-social support services • Set up standard operational procedures for integrated interprofessional care • Create clear communication and reporting lines • Organise regular information sharing • Strengthen the collaboration between the different teams by holding regular coordination meetings and clinical case management meetings with all involved (including patients) • Establish a patient pathway with internal and external referrals; facilitate referral back to the PHC after hospitalisation • Implement international clinical protocols for NCD patient management • Assess patients and raise their awareness on their physical and mental health condition and needs • Share responsibility with the patient to take an informed decision • Tailor health promotion messages by involving patients in the different sites • Assess the patient satisfaction with services
**Medicines and equipment**	• Subsidise medical products (medicines, diagnostic tools and devices, consumables, orthotic devices) • Work on supply chain to ensure availability of medical products • Align with national and international recommendations
**Health workforce**	• Specify roles and tasks within the job descriptions of the different team members and their reporting lines • Include interprofessional collaboration in regular feed-back given to the team members • Build capacity of the professionals by holding peer exchange meetings, providing access to trainings and continuous professional development • Empower team members • Give autonomy for participative decision making • Manage interpersonal conflict by having a solution-oriented approach and consulting the different parties involved
**Information systems and data sharing**	• Institute a unique patient identifier across the different health services within a register • Design a shared patient management tool (paper or electronic) including information on the presence of an NCD, mental health and psycho-social support or rehabilitation needs • Consider setting up a database for monitoring of patient outcomes and the interprofessional collaboration by integrating process and outcome indicators to improve quality of care and guarantee data protection and confidentiality
**Patient and Community Factors**	• Increase awareness on NCDs of the community • Enquire about patient’s capacities, priorities • Empower patients on setting priorities • Educate patients on self-management • Include patients and care givers in decisions and interprofessional team meetings • Get feedback from patients and care givers

## 5 Conclusions

Care for PLWNCDs is complex in all settings and requires collaboration of the different health care professionals and working beyond the silos of different health professions and services. In a country affected by a humanitarian crisis, in addition to ensuring lifesaving services for PLD (e.g. access and supply of medicines and diagnostic tools) we call for continuous support from the management of health organisations or institutions to interprofessional care for PLWNCDs. In this paper we develop a theoretical framework for interprofessional collaboration for PLD in a humanitarian organisation. The main factors favouring interprofessional collaboration are related to inclusive leadership, structural and organisational elements, interacting with the contribution of individual team members and the role and capacities of PLD. While leaders need to mitigate competition between professions and challenges linked to professional identities through mutual learning and showing consideration of all team members, efficient interprofessional collaboration requires resources and support in the reorganisation of work, commitment of the individual team members and consideration of the influence of the humanitarian and social context on PLD awareness of the disease, perceptions, needs and capacities. Providing interprofessional care to address the needs of PLWNCD in a humanitarian setting is feasible and requires strong commitment and collaboration of all involved.

## Funding

This research did not receive any specific grant from funding agencies in the public, commercial and non-profit sectors.

## CRediT authorship contribution statement

**Sigiriya Aebischer Perone:** Writing – original draft, Software, Methodology, Formal analysis, Conceptualization, Visualization, Project administration, Investigation, Data curation. **Kinda Khamasmie:** Validation, Conceptualization, Writing – review & editing, Project administration. **Ranim Doukki:** Validation, Conceptualization, Writing – review & editing, Project administration. **Claudine Dauby:** Validation, Conceptualization, Writing – review & editing, Project administration. **Catherine Savoy:** Validation, Writing – review & editing. **François Chappuis:** Validation, Writing – review & editing. **Nicolas Perone:** Writing – review & editing, Software, Conceptualization, Validation, Methodology. **David Beran:** Writing – review & editing, Supervision, Validation, Methodology.

## Declaration of competing interest

The authors declare that they have no known competing financial interests or personal relationships that could have appeared to influence the work reported in this paper.
